# Tobacco LSU-like protein couples sulphur-deficiency response with ethylene signalling pathway

**DOI:** 10.1093/jxb/ert309

**Published:** 2013-10-01

**Authors:** Grzegorz Moniuszko, Marek Skoneczny, Katarzyna Zientara-Rytter, Anna Wawrzyńska, Dawid Głów, Simona M. Cristescu, Frans J. M. Harren, Agnieszka Sirko

**Affiliations:** ^1^Institute of Biochemistry and Biophysics PAS, Pawinskiego 5A, 02-106 Warsaw, Poland; ^2^Life Science Trace Gas Facility, Molecular and Laser Physics, Institute of Molecules and Materials, Radboud University, Heyendaalseweg 135, The Netherlands

**Keywords:** Ethylene, response to nutrients deficit, sulphate, sulphur deficiency, tobacco, transcriptome, transgenic plants.

## Abstract

Most genes from the plant-specific family encoding Response to Low Sulphur (LSU)-like proteins are strongly induced in sulphur (S)-deficient conditions. The exact role of these proteins remains unclear; however, some data suggest their importance for plants’ adjustment to nutrient deficiency and other environmental stresses. This work established that the regulation of ethylene signalling is a part of plants’ response to S deficiency and showed the interaction between UP9C, a tobacco LSU family member, and one of the tobacco isoforms of 1-aminocyclopropane-1-carboxylic acid oxidase (ACO2A). Increase in ethylene level induced by S deficiency does not take place in tobacco plants with *UP9C* expressed in an antisense orientation. Based on transcriptomics data, this work also demonstrated that the majority of tobacco’s response to S deficiency is misregulated in plants expressing *UP9C*-antisense. A link between response to S deficiency, ethylene sensing, and LSU-like proteins was emphasized by changes in expression of the genes encoding ethylene receptors and F-box proteins specific for the ethylene pathway.

## Introduction

Plants utilize sulphate for the synthesis of primary and secondary compounds. Sulphate is metabolized in a series of steps including its activation and reduction. Unreduced sulphate can be used for the synthesis of sulphated compounds, while after reduction to sulphide it can be incorporated into cysteine (Lewandowska and Sirko, 2008; Davidian and Kopriva, 2010; Takahashi *et al.*, 2011). Sulphur (S) deficiency negatively affects the yield and development of crops (Scherer, 2001;;Haneklaus *et al.*, 2005), as well as their quality as a component of processed food (Muttucumaru *et al.*, 2006). Most data on gene expression in response to S nutrition status are available for *Arabidopsis thaliana* (Hirai *et al.*, 2003; Maruyama-Nakashita *et al.*, 2003; Nikiforova *et al.*, 2003). However, data for other species, such as poplar (Honsel *et al.*, 2012), wheat (microarray data are available in the ArrayExpress database, www.ebi.ac.uk/arrayexpress, under the accession number E-MEXP-1694), and tobacco (Lewandowska *et al.*, 2005; Wawrzynska *et al.*, 2005) also exist.

In *A. thaliana*, the LSU (Response to Low Sulphur) family includes four genes, *LSU1*–*LSU4*. *LSU1* (At3g49580) was indicated as an important connector in the gene–metabolite hormone-related network of response to S; however, no experimental work has focused on this gene (Nikiforova *et al.*, 2005). Products of *LSU1* and *LSU2* were identified as significantly targeted hubs among 165 putative effector targets of the effector proteins from two plant pathogens, and the enhanced disease susceptibility of an *lsu2* mutant confirmed the significance of this finding (Mukhtar *et al.*, 2011). Analysis of transcriptomic data sets for four different treatments, using a bioinformatic network-based approach, resulted in the identification of *LSU2* as one of the six genes whose products are putatively involved in the signalling crosstalk of nitrogen, iron, S, and phytohormones (Omranian *et al.*, 2012). LSU1 and LSU2 are highly interconnected and their cellular interactomes include proteins involved in various metabolic processes, such as DNA and RNA binding, redox homeostasis, and others (*Arabidopsis* Interactome Mapping Consortium, 2011). Besides, the level of *LSU1* transcript (but not other *LSU*s) is correlated with levels of *O*-acetylserine (OAS), a molecule previously speculated to be the sensor of S status (Hubberten *et al.*, 2012).

The tobacco LSU-like family includes at least six members (UP9A–UP9F), most of which are up-regulated by S deficiency. Tobacco AB3 lines (with silenced expression of *LSU*-like genes due to the overexpression of *UP9C* in the antisense orientation) were described previously (Lewandowska *et al.*, 2010). Analysis of these lines suggested that LSU-like proteins play a role in adjustment of plant metabolism to S deficiency at multiple levels.

Ethylene is a hydrocarbon gas that regulates many processes of plant growth and development. It also plays an important role in plants’ responses to environmental stresses (for reviews, see Adie *et al.*, 2007; Lin *et al.*, 2009), including nutritional stresses. Ethylene production was changed during phosphorus, potassium, calcium, magnesium, nitrogen, and iron deficiency (Lynch and Brown, 1997; Benlloch-Gonzalez *et al.*, 2010; Hermans *et al.*, 2010). The biosynthesis of ethylene is a two-step reaction. *S*-Adenosyl-l-methionine (SAM) is first converted to 1-aminocyclopropane-1-carboxylic acid (ACC) by ACC synthase (*S*-adenosyl-l-methionine methylthioadenosine-lyase, EC 4.4.1.14; ACS). Next, ACC is converted to ethylene by ACC oxidase (aminocyclopropanecarboxylate oxidase, EC 1.14.17.4; ACO). The *Arabidopsis* genome encodes 12 *ACS*- and 5 *ACO*-like genes (Lin *et al.*, 2009). The exact number of genes that encode ACS and ACO isoforms in tobacco is not known; however, at least four sequences for ACS (*NtACS1–NtACS4*) and at least three sequences for ACO (*NtACO1–NtACO3*) with complete open reading frames (ORFs) can be retrieved from the GenBank database. Ethylene signal transduction and ethylene biosynthesis involve multiple regulatory steps (Zhao and Guo, 2011). In *Arabidopsis*, the pathway starts with ethylene sensing by membrane-associated receptors (ETR1, ETR2, ERS1, ERS2, and EIN4). Next, the signal proceeds to ETHYLENE INSENSITIVE2 (EIN2) followed by ETHYLENE INSENSITIVE3 (EIN3) and ETHYLENE-INSENSITIVE3-LIKE 1 (EIL1), which is the border step between general and stimulus-specific types of ethylene responses (Li and Guo, 2007). Levels of EIN3 and EIL1 transcription factors are controlled by degradation in the 26S proteasome and depend on the SCF (EBF1/EBF2) complex of ubiquitin ligase.

A member of the EIL family, SULPHUR LIMITATION 1 (SLIM1/AtEIL3), which is not involved in ethylene response, was identified as a transcriptional regulator of S-deficiency response in *Arabidopsis* (Maruyama-Nakashita *et al.*, 2006). Moreover, direct binding of NtEIL2 (a tobacco member of the EIL family) to the *cis*-element (UPE-box) present in the *UP9C* promoter was shown, and NtEIL2 was demonstrated to be responsible for the S-deficiency-dependent induction of *UP9C* (Wawrzynska *et al.*, 2010). Additionally, a set of MYB factors that coordinate primary and secondary sulphate assimilation by affecting the expression of the genes encoding enzymes involved in the synthesis of secondary S metabolites (glucosinolates) and in reductive assimilation of sulphate have been identified (Yatusevich *et al.*, 2010).

It was previously concluded that UP9C is necessary for plants’ appropriate response to S deficiency (Lewandowska *et al.*, 2010). Here, it is shown that increased ethylene production is part of the tobacco plant’s response to S deficiency. It seems to be under the control of UP9C, possibly due to its direct interaction with ACO. This work also shows that appropriate levels of LSU-like proteins are necessary for the majority of tobacco transcriptome changes in response to S deficiency. This study speculates that LSU-like proteins modulate the ethylene signalling pathway and that ethylene perception plays an important role in the S-deficiency response.

## Materials and methods

### Plant cultures and media

AB medium, prepared according to a previously described protocol (Wawrzynska *et al.*, 2005), was used. Sulphur-deficiency medium (AB-S) was prepared by exchanging MgSO_4_ for an equimolar amount of MgCl_2_.

### Tobacco plant material and growth conditions

Construction of the transgenic AB3 lines (overexpressing *UP9C* in the antisense orientation) was described previously (Lewandowska *et al.*, 2010). The homozygous T2 generation of the AB3-1 and AB3-9 lines was used. The parental *Nicotiana tabacum* cv. LA Burley 21 (wild-type; WT; described in Legg *et al.*, 1970) was always used as a control. Tobacco seeds were surface sterilized before germination. All plants were grown under 60% relative humidity, with a 16/8 light/dark cycle (300 μmol photons m^–2^ s^–1^) at 23/19 °C. For the measurement of ethylene production, seeds were germinated in the containers (Araponics, SA, USA) and maintained in hydroponic conditions with the medium changed every week. Two days before ethylene measurement, 6-week-old tobacco plants were transferred into glass Erlenmeyer flasks filled with 25ml of fresh AB (nS conditions) or AB-S media (–S conditions). For the microarrays, plants were cultivated in hydroponics pots, with the medium changed every second week. The 8-week-old plants were transferred either onto AB-S or AB. After 2 days, shoots were harvested, directly frozen in liquid nitrogen, and stored at –80 °C.

### Gene cloning and plasmid construction

Conventional techniques were used for DNA manipulation and *Escherichia coli* transformation (Sambrook *et al.*, 1989). Gateway BP and LR recombination reactions were performed as described in the manufacturer protocols (Invitrogen, USA). The plant binary Gateway vectors have been described by Karimi *et al.* (2002). All plasmids were verified by restriction digests and/or DNA sequencing. The pDG9 plasmid (for the production of GST-UP9C fusion) was obtained by cloning of the *Bam*HI-*Xho*I fragment from pU9-ET [obtained by cloning of the 350-bp PCR product, amplified with primers listed in Table 1 and digested with *Bam*HI and *Hin*dIII, into pET28a (Novagen, USA)] into pGEX4T-1 (Promega, Poland). The pB5 plasmid (for UP9C-CFP fusion) was obtained by cloning of the 350-bp PCR fragment containing the ORF of *UP9C* amplified with the primers listed in Table 1 into pDONR221 (Invitrogen) and recombination into the plant binary vector pK7CWG2. The 940-bp fragment containing the ORF of ACO2 amplified from a *N. tabacum* cDNA library with primers listed in Table 1 was cloned into pENTR/D-TOPO vector (Invitrogen). The pGM1 and pGM2 plasmids for localization of the ACO2A and ACO2B fusions with yellow fluorescent protein (ACO2A-YFP and ACO2B-YFP) were prepared by recombination into the plant binary vector pH7YWG2 (Karimi *et al.*, 2002). The pACO2-ET, for the production of HIS-ACO2 fusion, was obtained by cloning of the 940-bp PCR product, amplified with primers listed in Table 1 and digested with *Bam*HI and *Hin*dIII, into pET28a (Novagen, USA). For the control reaction in pull-down analysis, pGEX4T-1 vector (Promega, Poland) expressing a GST tag was used.

**Table 1.** Primers used in this study

Name	Sequence (5′–3′)	Purpose
U9ATG	CGGGATCCATGTTTTCGACAATTGCT	Cloning *UP9C* into pET28a
U9stop	GCAAGCTTGGTACCTCATTGGGAACTGGGAAC	Cloning *UP9C* into pET28a
U9_gatef	GGGGACAAGTTTGTACAAAAAAGCAGGCTCAATGTTTTCGACAATTGCTGT	Cloning *UP9C* into pDONR221
U9_gater	GGGGACCACTTTGTACAAGAAAGCTGGGTCTCATTGGGAACTGGGAACGGT	Cloning *UP9C* into pDONR221 for N-terminal fusion possibility
ACOgWAYf	CACCATGGAGTTGCTTAACACTGAA	Cloning *ACO2* into pENTR/D-TOPO
ACOgWAYr	AACAGTAGCTATTGGGGCAG	Cloning *ACO2* into pENTR/D-TOPO for C-terminal fusion possibility
BAMiACOF	ACGGATCCATGGAGTTGCTTAACACTGAA	Cloning *ACO2* into pET28a
ECOiACOR	CCGAATTCATCAAAGTCTCAAACAGTAGC	Cloning *ACO2* into pET28a
aat2	GTACAAGAAAGCTGGGTCG	Reverse primer for checking the correctness of GATEWAY constructs

### Microarray analysis

Shoots from 10 individual plants of the same line grown under the same conditions were combined and powdered in liquid nitrogen. Each mix was made twice (using different plants), which provided two independent biological repetitions. Next, 100mg of the sample was used for RNA isolation with TRIZOL Reagent (Invitrogen) according to the manufacturer’s protocol. Then, RNA was purified using the RNeasy MinElute Cleanup Kit (Qiagen, Germany) and its quality and quantity were checked using the Agilent 2100 BioAnalyzer (Agilent Technologies, USA). Fluorescent cRNA was obtained using the Quick Amp Labelling Kit (Agilent Technologies). Two experimental variants of competitive hybridizations were carried out with the Tobacco Gene Expression Microarray, 4×44K (Agilent Technologies): (i) AB3-1 –S vs. AB3-1 nS; and (ii) WT –S vs. WT nS. Two-colour hybridizations, run in quadruplicates with dye swap between duplicates of the same variant, were performed according to the Agilent Two-Color Microarray-Based Gene Expression Analysis protocol. Immediately after washing, the slides were scanned using an Axon GenePix 4000B scanner (Molecular Devices, USA). Photomultiplier (PMT) gain was adjusted individually (between 550 and 750 volts) to obtain optimal images. Feature extraction was performed using GenePix Pro software (Molecular Devices).

### Microarray data analysis

Statistical analysis of Lowess-normalized data as well as calculation of *P*-values and log_2_ ratio values were performed using Acuity 4.0 (Molecular Devices) and Microsoft Office Excel (Microsoft, USA). Regulation of particular gene expression levels was inferred from the log_2_ ratio value. For comparison of transgenic and WT plants’ response to S deficiency (AB3 –S/AB3 nS vs. WT –S/WT nS), significant features were chosen based on a formula: *P*-value < 0.05 or *P*-value ≤ (–0.001*x* ^5^ + 0.0178*x* ^4^ – 0.1032*x* ^3^ + 0.2008*x* ^2^ – 0.2022*x* + 1.0771), where *x* = 20log_2_ ratio + 1. Such a method allowed not only the choosing of the reliable data for the regulated genes, but also for the unregulated ones, which permitted a good comparison of the data for the plant lines used. For further analysis, only the 15 681 genes that pass through the formula in both plant lines were chosen. After that, the genes with log_2_ ratio values over 0.5 or below –0.5 were extracted as up-regulated and down-regulated, respectively, and those 574 genes were used in Gene Ontology analysis, as described below. To extract more information about the chosen genes, additional log_2_ ratio values were calculated (AB3 nS/WT nS and AB3 –S/WT –S) based on raw fluorescence data for each channel. This technique is less reliable; however, the stability of fluorescence medians sum between the different arrays legitimate obtained results as at least valuable supplementary data. For cluster analysis, only the 472 genes that were annotated or had known *Arabidopsis* homologues were selected. The data were submitted to ArrayExpress database and are available with the accession number E-MEXP-3699 (http://www.ebi.ac.uk/arrayexpress).

### Yeast two-hybrid analysis

The yeast two-hybrid experiments were performed as described previously (Lewandowska *et al.*, 2010).

### Pull-down experiment

The presence of recombinant proteins GST-UP9C, HIS-ACO, and GST in the respective bacterial extracts were confirmed by immunoblots using rabbit polyclonal anti-GST IgG (Sigma-Aldrich, Poland) or anti-His IgG (Santa Cruz Biotechnology, USA) as primary antibody and anti-rabbit IgG conjugated to alkaline phosphatase (Sigma-Aldrich) as secondary antibody. The bacterial protein extract including recombinant His-tagged ACO was purified under denaturing (8M urea) conditions on His-select HF Nickel Affinity Gel (Sigma-Aldrich). After a five-step wash, with the last two steps using native buffer, protein extracts from bacteria producing GST-tagged UP9C or the GST tag without any fusion protein (as a control) were incubated with gel at 4 °C overnight with gentle agitation. Then, the proteins were purified by a five-step wash under non-denaturing conditions and eluted with 250mM imidazole. Next, denaturing polyacrylamide gel electrophoresis and protein blots were performed.

### Transient protein expression and confocal fluorescence microscopy

Binary plasmids containing *UP9C-CFP*, *ACO2A-YFP*, and *ACO2B-YFP* expression cassettes were introduced into *Agrobacterium tumefaciens* LBA4404. Subsequent steps of material preparation and confocal fluorescence microscopy observation were performed as described previously (Zientara-Rytter *et al.*, 2011).

### Ethylene measurement

Ethylene production was measured with a laser-based photoacoustic ethylene detector (type ETD-300, Sensor Sense, Nijmegen, the Netherlands) in line with a flow-through sampling system (type VC-6, Sensor Sense). The detector is able to measure in real time about 300 parts per trillion (10^12^) by volume within a 5-s time scale (Cristescu *et al.*, 2008). The single plant was placed into a 2-l glass cuvette that was hermetically closed and fitted with inlet and outlet ports. The sampling system set a continuous air flow at a constant rate of 3.0 l h^–1^ through the cuvettes containing the 6-week-old tobacco plants and alternately directed the gas flow of one cuvette to the detector. Ethylene production was measured in succession of 10 minutes for each cuvette.

Data were continuously collected for about 22 hours in constant light condition. The AB and AB-S media were used as blank controls. Every part of the equipment was checked for ethylene production with negative results. Ethylene production in parts per billion (10^9^) by volume is related to the ethylene emission rate, which was calculated by multiplying the measured value with the flow rate and divided by the fresh weight, resulting in nl h^–1^ (g FW)^–1^.

### Statistical analysis

SMART6 (Letunic *et al.*, 2009) was used for the identification of the protein domains and patterns; NetNES 1.1 (la Cour *et al.*, 2004) was used to predict leucine-rich nuclear export signals (NES); and Wolf PSORT (Horton *et al.*, 2007) was used to predict cellular location. Gene Ontology analysis was performed using AgriGO (Du *et al.*, 2010). Singular Enrichment Analysis (SEA) was performed with the Agilent tobacco genome array as reference/background. Chi-squared test was chosen as the statistical test method; the minimum number of mapping entries was set to five; Yekutieli (false discovery rate (FDR) under dependency) was used as the multitest adjustment method, with the significance level of 0.01.

The hierarchical clustering of differentially regulated genes was performed with Cluster 3.0 (de Hoon *et al.*, 2004) using the average linkage clustering algorithm and visualized by Java TreeView (Saldanha, 2004).

### Accession numbers

The sequences used in this study can be retrieved from GenBank using the following accession numbers: *UP9C* (AY547446), *ACO2* (X83229), *Joka31A* (GU066878), *Joka31B* (GU066879), *ACO2-A* (HQ418208), and *ACO2-B* (HQ418209).

## Results

### ACC oxidase interacts with UP9C

**Fig. 1.**
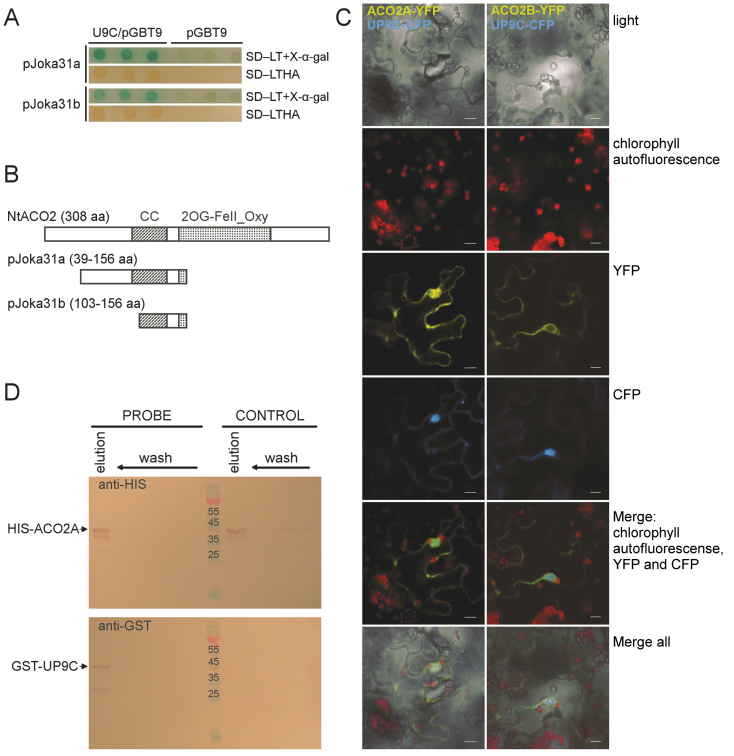
Interaction and co-localization of tobacco UP9C and NtACO2. (A) Growth of three independent colonies containing the ‘bait’ (UP9C/pGBT9) and ‘prey’ (pJoka31a or pJoka31b) plasmids. The ‘bait’ plasmid contains sequence encoding UP9C cloned into the pGBT9 vector enabling formation of the translational fusion with the DNA-binding domain of GAL4. The ‘prey’ plasmids contain sequences encoding fragments of NtACO2 found in yeast two-hybrid experiment using cDNA library from tobacco (present in the pGAL424 vector enabling formation of the translational fusion with sequence encoding activation domain of GAL4). Interaction between UP9C and the fragments of NtACO2 enables formation of an active GAL4 and reporter genes induction, which is monitored on two kinds of media, SD-LT+X-α-gal and SD-LTHA. The empty vector (pGBT9) was used as a negative control, where the lack of blue colour on SD-LT+ X-α-gal and the lack of growth on SD-LTH indicate that the reporter genes were inactive. (B) The scheme of NtACO2 protein and its fragments encoded by the inserts present in clones pJoka31a and pJoka31b; the numbers of amino acids and the location of a coiled-coil region (CC) and a domain characteristic for 2-oxoglutarate (2OG) and Fe(II)-dependent oxygenase superfamily (2OG-Fell_Oxy; PFAM Nr PF03171) are indicated. (C) Localization of transiently co-expressed UP9C-CFP and two variants of NtACO2-YFP in leaves of *Nicotiana benthamiana*; channels from the top are: visual light, chlorophyll autofluorescence, YFP, CFP, merge of fluorescence channels, merge of all. (D) Interaction between UP9C and NtACO2A shown in pull-down experiment. The His-tagged ACO2A bound to the Nickel Affinity Gel was used as a probe and its interaction with GST-tagged UP9C (PROBE) and lack of interaction with GST without any fusion protein (CONTROL) was verified. The His-tagged ACO2A and the GST-tagged UP9C were detected by the polyclonal antibodies recognizing the respective tags. The details are described in Materials and Methods.

It was previously reported that the tobacco protein UP9C has many potential interactors (Lewandowska *et al.*, 2010; Zientara-Rytter *et al.*, 2011). In this work, taking into consideration the number of clones encoding ACO (13 out of 33 clones found in the yeast two-hybrid assay), the ACO-UP9C interaction was verified. In fact, two types of ACO clones were frequently identified, pJoka31a and pJoka31b (Fig. 1A–B). Both clones contain inserts which exhibit sequence similarity to *ACO2* from Samsun NN tobacco (accession number X83229), and the insert in pJoka31b is a part of the insert present in pJoka31a (Fig. 1B). The cDNA sequence of *NtACO2* was amplified (with primers designed to bind to its 3′ and 5′ ends and based on X83229) using a cDNA library from LA Burley 21, which had been a source of cDNA for the yeast two-hybrid experiment (Lewandowska *et al.*, 2010). Two sequences containing complete ORFs were identified, *NtACO2A* and *NtACO2B*. Neither of them were identical to the *NtACO2* sequence in databases, yet *NtACO2A* revealed 99.8% identity. Besides, the fragment of ACO protein encoded by *Joka31B* was identical to the corresponding part of the protein encoded by *NtACO2A*. The fragment of ACO interacting with UP9C overlaps with two features predicted during *in silico* analysis of the ACO2 protein, a coiled coil region (92–132 aa) and a leucine-rich NES (118–130 aa). The predicted subcellular location of the ACO2 protein is cytosolic, while UP9C is present mainly in the nucleus, but also in the cytoplasm (Lewandowska *et al.*, 2005, 2010).

To show the possibility that these proteins can interact within plant cells, plasmids with plant expression cassettes containing the coding regions of *UP9C* and either *ACO2A* or *ACO2B* in frame with the coding regions of the fluorescent proteins were prepared. The location of the fusion proteins was monitored in the leaf tissue of transiently transformed *Nicotiana benthamiana* plants. The ACO2B-YFP was present exclusively in the cytoplasm, while ACO2A-YFP and UP9C-CFP proteins co-localized in a similar double (nuclear and cytosolic) location (Fig. 1C). Additionally, the UP9C-ACO2A interaction was confirmed in vitro by the pull-down experiment using proteins produced in bacteria (Fig. 1D). The interaction between UP9C and ACO2B was not further investigated.

### Ethylene level increases during short-period sulphur deficiency in WT but not in *UP9C*-antisense lines of tobacco AB3-1 and AB3-9

**Fig. 2.**
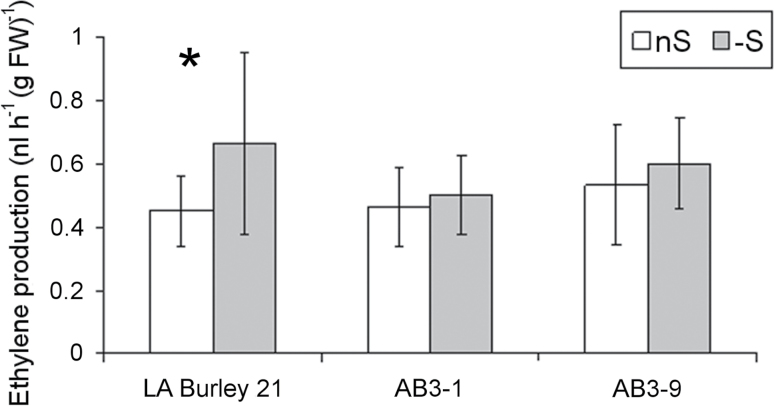
Ethylene production by LA Burley 21 (wild-type) and *UP9C*-antisense lines (AB3-1 and AB3-9) tobacco plants. Six-week-old plants were grown hydroponically under optimal conditions and transferred for 2 days into either S-sufficient (nS) or S-deficient (–S) media. A significant difference between nS and –S conditions (*t*-test; *P* < 0.05), marked by an asterisk was observed only in LA Burley 21 (wild type) plants; *n* = 11 for LA Burley 21, *n* = 7 for AB3-1, *n* = 3 for AB3-9.

Next, ethylene production in individual plants of the tobacco lines AB3-1 and AB3-9 (with silenced expression of *LSU*-like genes due to overexpression of *UP9C* in an antisense orientation, described in Lewandowska *et al.*, 2010), and of their parental line LA Burley 21 (WT) was monitored. Two days before the measurement, 6-week-old plants grown in S-sufficient conditions (nS) were transferred to either nS or S-deficient conditions (–S). One series of the experiment lasted 22–23 hours and included multiple (26–30) data points collected for each of the plant placed individually into the gas-tight container as described in Materials and Methods. The means from all except the first data points of the experiments were calculated. The results shown in Fig. 2 clearly indicate that in sulphur-deficient conditions the parental plants (LA Burley 21) produced more ethylene (140%) than in sulphur-sufficient conditions. In contrast, no statistically significant sulphur-deficiency-induced increase of ethylene production was observed in AB3 lines. In –S conditions, the ethylene production by AB3-1 and AB3-9 was reduced to 75% and 90%, respectively, of that by the parental line maintained in the same conditions. Interestingly, in nS conditions the AB3-9 line produced slightly more ethylene (120%) than the parental line. This observation suggested that UP9C is required for the increased production of ethylene during S deficiency and raised the question of the effect of *UP9C* silencing on gene expression profile during the plants’ response to S deficiency.

### Changes in the transcriptome of the AB3-1 line

**Fig. 3.**
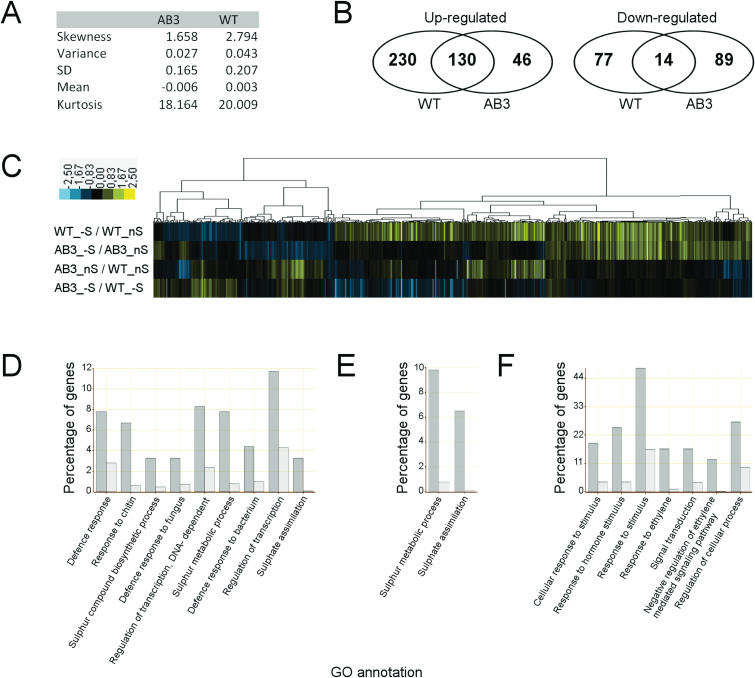
Microarray analysis of S-deficiency response in AB3-1 and wild-type (WT) tobacco plants. Eight-week-old plants were grown hydroponically under optimal conditions and transferred for 2 days into either S-sufficient (nS) or S-deficient (–S) media. The experiment was performed in duplicate, using shoots of 10 individual plants for each condition. Statistical analysis of microarray data (A) was limited to 15,681 genes with reliable expression in both lines (for the criteria of selection, see Materials and Methods). Venn diagrams (B) and heat-maps (C) of the regulated genes are shown for both lines. The genes included in analysis were chosen based on the log_2_ ratio of spot intensity, using the arbitrary limits of ≥0.5 and ≤–0.5), for up- and down-regulation by S-deficiency genes, respectively. Over-representation of Gene Ontology (GO) entries among genes up-regulated by S deficiency in WT (D) as well as AB3 (E), and among genes down-regulated by S deficiency in AB3-1 (F). The dark-grey and light-grey columns indicate the percentage of genes from the indicated category in the input (analysed) and the reference (background; all genes in the microarrays) lists, respectively.

To compare transcriptomic responses to sulphur deficiency only line AB3-1 was selected, as both *UP9C-antisense* lines showed similarly disturbed ethylene production during –S conditions. Eight-week-old plants were grown hydroponically in nS medium and transferred for 2 days into either nS or –S. Transcripts were analysed in shoots and the effects of S deficiency on gene expression profile in AB3-1 were compared with the response in WT. Analysis of data started with selecting genes with reliable (see Materials and Methods for explanation) expression in both lines. Comparison of the statistical characteristics of the distributions of extracted log ratios (reflecting the expression levels) in both lines, especially larger skewness for WT distribution, suggested that higher regulation of gene expression in response to S deficiency could be expected in WT than in AB3-1 (Fig. 3A). Accordingly, the total number of regulated genes was higher in WT than in AB3-1 (Fig. 3B). Only 130 of 360 genes up-regulated by S deficiency in WT were also up-regulated in AB3-1, and only 14 of 91 genes down-regulated in WT were also down-regulated in AB3-1. There were also genes regulated only in AB3-1 but not in WT (46 and 89 up- and down-regulated, respectively).

Visualization of the effect of S deficiency on gene expression profiles (of 472 genes with either known *Arabidopsis* homologue or any description) in both lines revealed more information (Fig. 3C). In Fig. 3C, the genes are clustered hierarchically to show from the right-hand side genes that were up-regulated in both lines in response to S deficit, and next the genes that were up-regulated in WT but not regulated in AB3-1. The difference in these genes between WT and AB3-1, in response to S deficit, resulted from a difference between AB3-1 and WT in either nS or –S conditions. Next are clusters containing genes that were down-regulated in both lines, down-regulated in the AB3-1 line and in WT, respectively. Most genes identified as down-regulated by –S in AB3-1 but not in WT had higher expression in AB3-1 than in WT under nS conditions.

Deregulation of particular genes in AB3-1 in comparison with WT could be an indicator of LSU/UP9 involvement in certain processes. The gene sets specified in Fig. 3B were further classified into Gene Ontology (GO) categories. The GO analysis tool could help in the identification of over-represented categories of genes. The set of 360 genes up-regulated in –S in WT was automatically categorized into 23 over-represented processes, including the two common with processes over-represented by genes induced by –S in AB3-1, namely sulphur metabolism and sulphate assimilation (as will be discussed in more detail), although they could be manually rearranged into a lower number of non-redundant GO processes (Fig. 3D). Apart from ‘sulphur metabolic process’ (14 genes), including ‘sulphate assimilation’ (six genes) and ‘sulphur compound metabolic process’ (six genes), more categories of genes could be distinguished, including ‘regulation of transcription’ (21 genes) with its ‘DNA-dependent’ part (15 genes), ‘defence response’ (14 genes) with parts dedicated to ‘defence response to fungi’ (six genes) and ‘defence response to bacteria’ (eight genes), and ‘response to chitin’ (12 genes). Many of these groups contain some ethylene-related genes, such as homologues of *Arabidopsis ERF1*, *ERF2*, and *ACO*; however, not all ethylene indicators were induced by S deficiency in WT. The expression of the genes classified as being involved in ‘response to stimulus’ was elevated by S deficiency only in WT but not in AB3-1. Significant enrichment of only two GO processes, namely the ‘sulphur metabolic process’ [represented by nine genes encoding isoforms of *S*-adenosylmethionine synthetase (SAMS) and APS reductase (APR)] and the ‘sulphate assimilation process’ (represented by six genes encoding isoforms of APR), were over-represented within the set of 176 genes up-regulated by –S in AB3-1 (Fig. 3E). GO analysis of the set of 46 genes up-regulated only in AB3-1 and the set of 91 genes down-regulated by S deficiency in WT failed to identify any enriched category. In contrast, 103 genes down-regulated by S deficiency in AB3-1 were over-represented in several GO processes (Fig. 3F), such as ‘response to stimulus’ (nine genes), ‘response to hormone stimulus’ (12 genes), ‘response to ethylene’ (eight genes), ‘regulation of cellular process’ (13 genes), and ‘signal transduction’ (eight genes). Two genes from the last category were recognized as homologues of *Arabidopsis* genes, encoding proteins involved in abscisic acid- and cytokinin-mediated signalling pathways (ATHB-7 and AHP1, respectively). The other six genes in that category belong to the substantially over-represented ‘negative regulation of ethylene signalling pathway’ GO process. These six genes were classified as homologues of *Arabidopsis* EBF1, EIN4, and ERS1.

## Discussion

The regulation of plants’ response to S deficiency takes place on many levels, yet the regulation of transcription is still postulated to be responsible for most of the final effects (Lewandowska and Sirko, 2008). In this work, it has been shown that the up-regulation of genes classified into multiple GO processes by S deficiency is dependent on LSU-like proteins (Fig. 3). In fact, from all genes of GO processes up-regulated by S deficiency in WT, only those encoding SAMS and APR isoforms (classified into two GO processes, namely ‘sulphate assimilation’ and ‘sulphur metabolic process’) and two additional genes with products involved in cell redox homeostasis and oxidative damage repair were up-regulated in AB3-1 like in WT. These data suggest that LSU-like proteins function as regulators of S-deficiency response; however, there is a part of the S-deficiency response (S flux through reductive assimilation) that is not controlled by them.

The question of the relationship between S supply and ethylene biosynthesis has been addressed previously. Increased ethylene biosynthesis during the response of excited tomato roots to S deficiency was observed (Zuchi *et al.*, 2009). However, an invasive sample treatment (basically the wounding stress) might influence ethylene biosynthesis by itself, independently from S supply. Besides, a longer period of nutrient deficiency (5 days compared with 2 days in the current work) might lead to problems with distinguishing between direct and indirect effects of S deficiency. These data indicate that tobacco plants increase ethylene production in response to short-term S deficiency (Fig. 2). Recently, links of S metabolism with ethylene were discussed in work dealing with the response of mustard to cadmium (Cd) (Masood *et al.*, 2012). The authors noticed a similarity between ethephon treatment and increased S supply in alleviation of the toxic effects of Cd. The ethylene biosynthesis inhibitor 1-aminoethoxyvinylglycine (AVG) reversed the effects of S surplus. Cd stress results in high demand for S metabolites, which is equivalent to S-deficiency stress. Thus, these results are in agreement with the requirement for ethylene during the response to Cd stress.

LSU-like proteins might function as modulators of ethylene biosynthesis in –S conditions, affecting either the function or stability of the enzymes involved in ethylene synthesis. Most experimental data suggest that ACS is the key enzyme in regulation of the pathway (Chae and Kieber, 2005; Argueso *et al.*, 2007). However, in some cases ACO may be a limiting factor (reviewed in Argueso *et al.*, 2007). The levels of *ACO* transcripts are regulated by ethylene itself and by other phytohormones (Lin *et al.*, 2009). According to STRING 8.3 output (Jensen *et al.*, 2009), no proteins interacting with *Arabidopsis* ACOs have been reported yet. The present results strongly suggest that LSU-like proteins directly interact with ACO and facilitate increased ethylene production during plants’ response to S deficiency. It is tempting to speculate that this interaction might play a role in ACO stability. No data on ACO degradation have been published yet. The significance of the detected UP9C interactions is unclear and need to be investigated further.

The complexity of the ethylene signalling cascade allows regulation at many levels. The UP9C does not influence the level of transcription of the genes encoding proteins of the EIL family; however, it is known that they are rather regulated by protein stability (Zhao and Guo, 2011). Interestingly, expression of the genes encoding homologues of *Arabidopsis* EBF1 and tomato EBF2, involved in proteasome-mediated degradation of EIN3 in the absence of ethylene, was strongly misregulated in AB3-1. It was lower under S-deficient than in S-sufficient conditions, while in WT no significant changes in *EBF1* and *EBF2* expression were observed. The similar pattern of changed regulation in AB3-1 was observed for genes encoding homologues of *Arabidopsis* EIN4 and ERS1. These proteins represent two subfamilies of ethylene receptors (Wang *et al.*, 2006). It can be speculated that LSU-like proteins modulate the ethylene signalling pathway and that ethylene perception plays an important role in the S-deficiency response.

Results of the analysis of *slim1* mutants suggested that the transcription factor SLIM1/EIL3 was the main regulator of the response to S deficiency in *A. thaliana* (Maruyama-Nakashita *et al.*, 2006). The present data suggest that LSU-like proteins are needed for SLIM1-dependent regulation and possibly for the response to S deficiency, regulated by other hypothetical transcription factors. S-dependent induction of transcripts corresponding to *LSU* family members was lower in *Arabidopsis slim1* mutants than in WT, but was still detectable. It implies that SLIM1 and other factors that remain to be identified jointly regulate the transcription of *LSU*-like genes under –S conditions.

**Fig. 4.**
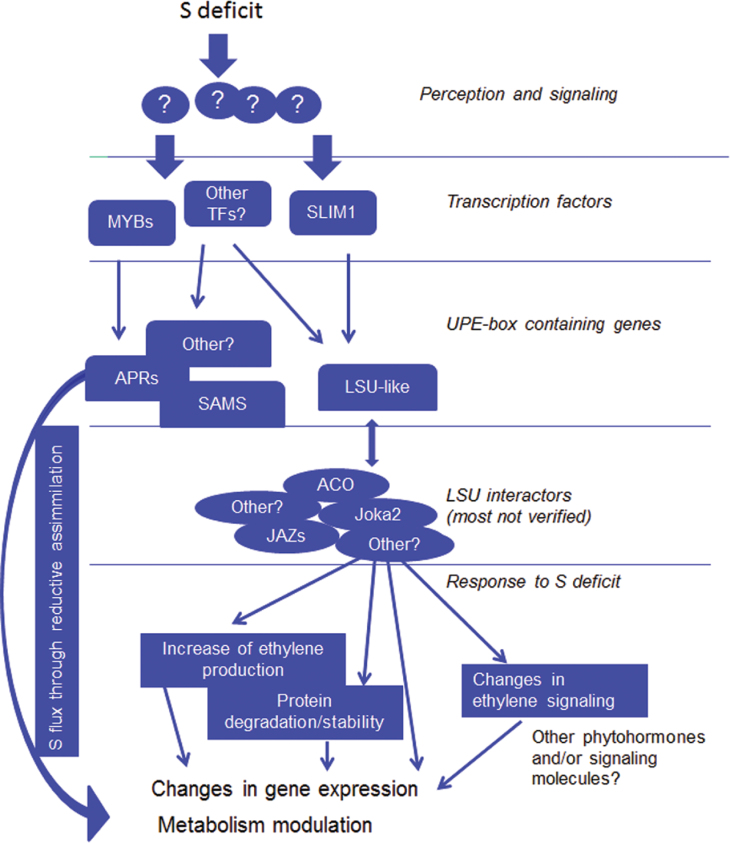
Model of the role of LSU/UP9 proteins in plants’ response to S deficiency (this figure is available in colour at *JXB* online).

A model describing the role of LSU-like proteins in plants’ response to S deficiency could be proposed (Fig. 4). Molecules speculated to be S status sensors, such as sulphate, OAS, and glutathione have been omitted. However, they are clearly upstream of LSU/UP9 proteins. Notably, it was recently shown that in *Arabidopsis*, the level of *LSU1* transcript was correlated with OAS (Hubberten *et al.*, 2012). However, levels of other *LSU* transcripts were not found to be correlated with OAS. On the other hand, LSU-like proteins must act downstream of the ‘primary’ transcription factors that are specific for plants’ response to S deficiency (such as SLIM1 or possibly MYB). More than one transcription factor must be involved in the regulation of *LSU*-like and other genes induced by S deficiency. Data from this study combined with previous results (Lewandowska *et al.*, 2010; Zientara-Rytter *et al.*, 2011) show that LSU-like proteins are responsible for tuning up gene expression and metabolite profiles in response to S deficiency, by influencing ethylene signalling and ethylene production. In addition, the influence of LSU/UP9 proteins on the activity and/or stability of proteins involved in the signalling pathways and synthesis of other hormones is quite possible, considering the long list of potential partners of LSU-like proteins. The molecular function of LSU-like proteins and their possible multiple interactions must be further investigated, and more research is needed in order to fully understand plants’ response to S deficiency and its multilevel regulation.
